# Effects of rasagiline on Parkinson’s Disease Questionnaire (PDQ-39) emotional well-being domain in patients with Parkinson’s disease: A post-hoc analysis of clinical trials in Japan

**DOI:** 10.1371/journal.pone.0262796

**Published:** 2022-01-25

**Authors:** Nobutaka Hattori, Atsushi Takeda, Yuki Hanya, Tadayuki Kitagawa, Masaki Arai, Yoshihiko Furusawa, Hideki Mochizuki, Masahiro Nagai, Ryosuke Takahashi

**Affiliations:** 1 Department of Neurology, Juntendo University Faculty of Medicine, Tokyo, Japan; 2 Department of Neurology, National Hospital Organization Sendai Nishitaga Hospital, Miyagi, Japan; 3 Department of Cognitive & Motor Aging, Tohoku University Graduate School of Medicine, Miyagi, Japan; 4 Japan Medical Office, Takeda Pharmaceutical Company Limited, Tokyo, Japan; 5 Japan Development Center, Takeda Pharmaceutical Company Limited, Osaka, Japan; 6 Department of Neurology, Osaka University Graduate School of Medicine, Osaka, Japan; 7 Clinical Research Support Center, Ehime University Hospital, Ehime, Japan; 8 Department of Neurology, Kyoto University Graduate School of Medicine, Kyoto, Japan; IRCCS E. Medea, ITALY

## Abstract

**Background:**

Identifying the factors that influence health-related quality of life (HRQoL) is of great scientific interest, but a potential causal relationship between treatment and HRQoL has yet to be fully elucidated. Japanese patients reported better HRQoL outcomes on the Parkinson’s Disease Questionnaire (PDQ-39) emotional well-being domain, a 6-question subset of the PDQ-39 which is considered to reflect the emotional aspects of the disease-specific HRQoL, when treated with rasagiline, than placebo, in both a monotherapy clinical trial (NCT02337725) and an adjunctive therapy clinical trial in patients with wearing-off phenomena (NCT02337738).

**Objective:**

To investigate how rasagiline exerts its effect on the PDQ-39 emotional well-being domain in Japanese patients with Parkinson’s disease.

**Methods:**

A path analysis was performed to assess the direct treatment effects of rasagiline on the PDQ-39 emotional well-being domain and the effects mediated indirectly through the influence on items related to motor symptoms by a post-hoc analysis of two clinical trials in Japan.

**Results:**

In the monotherapy trial, the PDQ-39 emotional well-being domain was mainly affected indirectly through items related to motor symptoms (80.7%) composed of the Movement Disorder Society-Unified Parkinson’s Disease Rating Scale (MDS-UPDRS) Part II (67.2%) and Part III (13.5%). In the adjunctive therapy trial, the PDQ-39 emotional well-being domain was also mainly influenced indirectly through effects on items related to motor symptoms (1 mg/day: 54.7%, 0.5 mg/day: 57.6%) composed of MDS-UPDRS Part II (1 mg/day: 35.6%, 0.5 mg/day: 40.9%), Part III (1 mg/day: 8.0%, 0.5 mg/day: 8.3%) and mean daily OFF-time (1 mg/day: 11.1%, 0.5 mg/day: 8.4%).

**Conclusions:**

The effects of rasagiline on the PDQ-39 emotional well-being domain were mediated primarily by influence on the subjective aspects of motor experiences of daily living.

## Introduction

Parkinson’s disease (PD) is a neurodegenerative disorder characterized by the loss of dopaminergic neurons in the substantia nigra pars compacta, which is classically characterized by symptoms such as bradykinesia, muscular rigidity, rest tremor, and postural and gait impairment [1]. In Japan, the estimated prevalence of PD is 100–150 per 100,000 people. Although incidence has remained stable, prevalence is increasing alongside the average population age and lifespan [2–6].

PD cannot currently be cured, so treatment has historically been aimed at alleviating motor symptoms [1]. Rasagiline, a selective and irreversible inhibitor of monoamine oxidase B [7, 8], has been shown to be efficacious at relieving the symptoms of PD and is well-tolerated when administered as monotherapy, or as an adjunct to levodopa, in Japanese patients with PD in two randomized, double-blind, placebo-controlled, 26-week studies [9, 10]. In these studies, significant improvements in total and subscale scores were recorded using a validated Japanese translation of the Movement Disorder Society-Unified Parkinson’s Disease Rating Scale (MDS-UPDRS) [9–11].

Previously, the management of motor symptoms was considered to be the most important treatment outcome for patients with PD, but health-related quality of life (HRQoL) is increasingly recognized as an important endpoint in clinical research involving patients with chronic conditions and an important outcome measure in their care [12, 13]. The self-reported 39-item Parkinson’s Disease Questionnaire (PDQ-39) [14] was used to assess HRQoL in the clinical trials of rasagiline in Japan [9, 10]. Notably, those trials presented the novel finding that rasagiline treatment for 26 weeks resulted in maintained baseline scores in the PDQ-39 emotional well-being domain, which is considered to reflect the emotional aspects of the disease-specific HRQoL and consists of 6 items (depressed, isolated/lonely, weepy/tearful, angry/bitter, anxious, and worried about the future) in both monotherapy in early PD, and adjunctive therapy in patients with PD and wearing-off phenomena with statistical significance compared to the placebo group. Moreover, a deterioration, which approached the threshold of being considered clinically meaningful, was observed in patients randomized to placebo, suggesting a clinically meaningful treatment effect of rasagiline on this domain [9, 10, 15]. Considering that the PDQ-39 emotional well-being domain has been reported to be related to mood measures such as depression and anxiety [16], and the prevailing scientific attention to rasagiline’s effect on non-motor symptoms in PD [17–19] (symptoms which may be partially related to dopamine deficiency [20]), it is worth elucidating how rasagiline exerted the effect on the PDQ-39 emotional well-being domain detected in the prior trials in Japan.

Identifying treatment-related factors that influence HRQoL is of great scientific interest and accumulating evidence has suggested that non-motor symptoms and other aspects of PD, such as the impact of PD on activities and/or experiences of daily living, may be more closely associated with HRQoL than objective assessments of motor symptoms [21–31]. However, the design of these studies was cross-sectional, and did not focus on the effects of individual pharmacological treatments on HRQoL.

Elucidating the mechanisms by which individual pharmacological treatments exert their effects on HRQoL domains could have a meaningful impact on clinical practice for patients with PD. However, a greater understanding of the role of individual treatments on HRQoL domains requires the assessment of potential causal relationships between interventions and outcome measures, and there is little published research focusing on PD in this area [32].

This post-hoc analysis aimed to investigate the direct and indirect effects of rasagiline on the emotional well-being domain of the PDQ-39 in Japanese patients with PD by applying path analyses to evaluate the extent to which motor symptom-related items, the traditional focus of treatment for PD, mediate any effect of rasagiline on the emotional aspects of HRQoL.

## Materials and methods

### Patients

This paper considered two randomized, double-blind, placebo-controlled studies of the efficacy and safety of rasagiline for 26 weeks in Japanese patients: [9, 10] A monotherapy trial in early PD (Phase 3, ClinicalTrials.gov identifier: NCT02337725) and an adjunctive therapy trial in patients with PD and wearing-off phenomena (Phase 2/3, ClinicalTrials.gov identifier: NCT02337738). Inclusion and exclusion criteria for these studies have been described previously [9, 10]. In brief, the monotherapy trial included patients aged 30–79 years with PD diagnosed within the past 5 years, an MDS-UPDRS Part II + III total score of ≥14, and at modified Hoehn and Yahr stages 1–3. Key exclusion criteria included a Mini-Mental State Examination (MMSE) score of ≤24; the subject having known or a history of schizophrenia, major or severe depression, or any other clinically significant psychiatric disease; and concomitant use of antidepressants. In the adjunctive trial, patients aged 30–79 years with PD were eligible, and all patients had been continuously receiving levodopa and a dopa decarboxylase inhibitor for at least 6 months before the run-in period and were experiencing wearing-off phenomena. At baseline, eligible patients had a modified Hoehn and Yahr stage score of 2–4 in the OFF state, and mean OFF time of ≥2.5 h per day. Among the exclusion criteria in the adjunctive trial were severe dyskinesia; an MMSE score of ≤24; the subject having known or a history of schizophrenia, major or severe depression, or any other clinically significant psychiatric disease; and concomitant use of antidepressants. Accordingly, the duration of PD was longer, and severity of disease greater, among participants in the adjunctive therapy study (Table 1) [9, 10]. Continued concomitant treatment with other treatments for PD, including dopamine agonists, anticholinergic drugs, amantadine, droxidopa, istradefylline, or zonisamide, was permitted in the adjunctive therapy study, provided the dose had not changed in the 14 days prior to starting the run-in period [10]. Entacapone was permitted provided that no dose alteration occurred after starting run-in and dose modification only occurred if the dose of levodopa was modified between weeks 0 and 6 [10]. Levodopa dose could be reduced if needed, due to adverse events, but alterations were not permitted after Week 6 [10].

**Table 1:** Baseline demographics and clinical characteristics.

Characteristic	Monotherapy trial in early PD		Adjunctive therapy trial in PD patients with wearing-off phenomena		
	Placebo	Rasagiline	Placebo	Rasagiline	Rasagiline
	(n = 126)	1 mg/day	(n = 141)	0.5 mg/day	1 mg/day
		(n = 118)		(n = 134)	(n = 129)
Age, years, mean (SD)	65.4 (8.81)	67.4 (8.96)	66.3 (7.62)	66.1 (8.74)	65.8 (8.48)
≥65 years, n (%)	79 (62.7)	79 (66.9)	93 (66.0)	86 (64.2)	80 (62.0)
Male gender, n (%)	54 (42.9)	53 (44.9)	53 (37.6)	58 (43.3)	46 (35.7)
Duration of PD, years; mean (SD)	1.56 (1.237)	1.97 (1.972)	8.90 (4.465)	8.53 (4.774)	9.49 (4.992)
≥10 years, n (%)	n/a	n/a	48 (34.0)	42 (31.3)	56 (43.4)
Modified Hoehn and Yahr stage, mean (SD)	2.15 (0.615)	2.18 (0.626)			
ON state			2.44 (0.608)	2.45 (0.542)	2.51 (0.566)
OFF state			3.22 (0.708)	3.25 (0.674)	3.30 (0.651)
MDS-UPDRS score, mean (SD)^a^					
Part I total	5.7 (3.58)	5.5 (3.83)^b^	8.7 (4.74)	9.0 (4.91)^d^	9.3 (4.82)^f^
Part II total	7.0 (4.64)	7.2 (5.47)^b^	13.0 (7.29)	13.7 (6.65)^d^	14.1 (7.23)^f^
Part III total	26.8 (11.59)	27.2 (13.80)^b^	26.8 (13.99)	28.7 (13.28)^d^	27.5 (13.09)^f^
Part IV total	0.0 (0.53)	0.0 (0.00)^b^	6.4 (2.66)	6.4 (2.55)^d^	6.7 (2.52)^f^
PDQ-39 emotional well-being score, mean (SD)^a^	16.90 (17.796)	12.65 (14.899)^b^	23.30 (18.665)^c^	22.79 (18.406)^e^	24.61 (18.525)
Duration of wearing-off phenomenon, years; mean (SD)	n/a	n/a	2.94 (2.869)	2.89 (2.749)	3.27 (2.990)
Daily OFF-time, hours; mean (SD)^a^	n/a	n/a	6.05 (2.278)	6.33 (2.562)^d^	6.12 (2.430)
Proportion of daily OFF-time, %; mean (SD)^a^	n/a	n/a	36.86 (13.373)	38.68 (14.278)^d^	36.89 (13.490)
Duration of levodopa use, years; mean (SD)	n/a	n/a	6.49 (4.402)	5.94 (3.984)	7.17 (4.800)
Levodopa total daily dose, mg; mean (SD)^a^	n/a	n/a	399.3 (141.03)^c^	407.8 (134.15)^e^	420.7 (166.42)
Levodopa frequency per day; mean (SD)^a^	n/a	n/a	3.8 (1.07)^c^	3.9 (1.32)^e^	4.1 (1.38)
Concomitant therapy, n (%)^a^					
COMT inhibitors	n/a	n/a	54 (38.3)	59 (44.0)	54 (41.9)
Dopamine agonists	n/a	n/a	122 (86.5)	106 (79.1)	114 (88.4)
Amantadine	n/a	n/a	23 (16.3)	26 (19.4)	33 (25.6)
Anticholinergics	n/a	n/a	12 (8.5)	13 (9.7)	13 (10.1)
Droxidopa	n/a	n/a	8 (5.7)	11 (8.2)	11 (8.5)
Istradefylline	n/a	n/a	33 (23.4)	24 (17.9)	35 (27.1)
Zonisamide	n/a	n/a	45 (31.9)	51 (38.1)	52 (40.3)

The data shown are for all randomized patients.

^a^At the end of the run-in period.

^b^n = 117.

^c^n = 139.

^d^n = 133.

^e^n = 132.

^f^n = 128.

COMT, catechol-O-methyltransferase; MDS-UPDRS, Movement Disorder Society-Unified Parkinson’s Disease Rating Scale; n/a, not applicable; PD, Parkinson’s disease; PDQ-39: 39-item Parkinson’s disease questionnaire; SD, standard deviation.

Institutional review board approval was obtained at each study site, and all patients provided written, informed consent prior to enrollment. Both studies were conducted in accordance with the ethical principles of the Declaration of Helsinki, the International Conference on Harmonization unified guidelines and local regulations. Full details of study methodologies and patient characteristics have been published previously [9, 10].

### Measures

Two instruments were used to quantify the condition of the patients included in this study: the MDS-UPDRS and the PDQ-39. Participants in the adjunct therapy trial also completed a home dairy to track their OFF time. The MDS-UPDRS comprises four subscales (Parts) assessing all elements of PD symptomatology. The Parts are: Part I (non-motor aspects of experiences of daily living), Part II (motor aspects of experiences of daily living); Part III (motor examination); and Part IV (motor complications). Part I comprises 6 items assessed in a semi-structured interview and 7 self-reported items, Part II comprises 13 self-reported items, Part III includes 18 items comprising 33 individual measurements, and Part IV comprises 6 items assessed in a semi-structured interview [33]. Total scores are summed from the item scores in each MDS-UPDRS Part (I-IV). All items are scored from 0–4, with 0 representing a normal score and 4 representing severe impairment.

Patients used their home diary to record OFF-time at 30-minute intervals for 24 hours during the 7 days preceding the designated weeks visits in the adjunctive therapy trial [10].

The PDQ-39 is entirely self-reported by patients and assesses HRQoL [14]. As the name implies, the PDQ-39 comprises 39 items divided into 8 different domains. Of these 39 items, 6 (items 17–22) pertain to emotional well-being and comprise depressed, isolated/lonely, weepy/tearful, angry/bitter, anxious, and worried about the future. Respondents rate each item on a scale from 0 (never) to 4 (always), reflecting how often they experienced the accompanying characteristic. All items’ scores are summed, divided by the maximum possible score, and multiplied by 100 to achieve a total score, with higher scores indicating more significantly impaired HRQoL. Aside from the emotional well-being domain, the other 7 domains comprise mobility, activities of daily living, stigma, social support, cognition, communication and bodily discomfort.

### Path analysis

**Fig 1:**
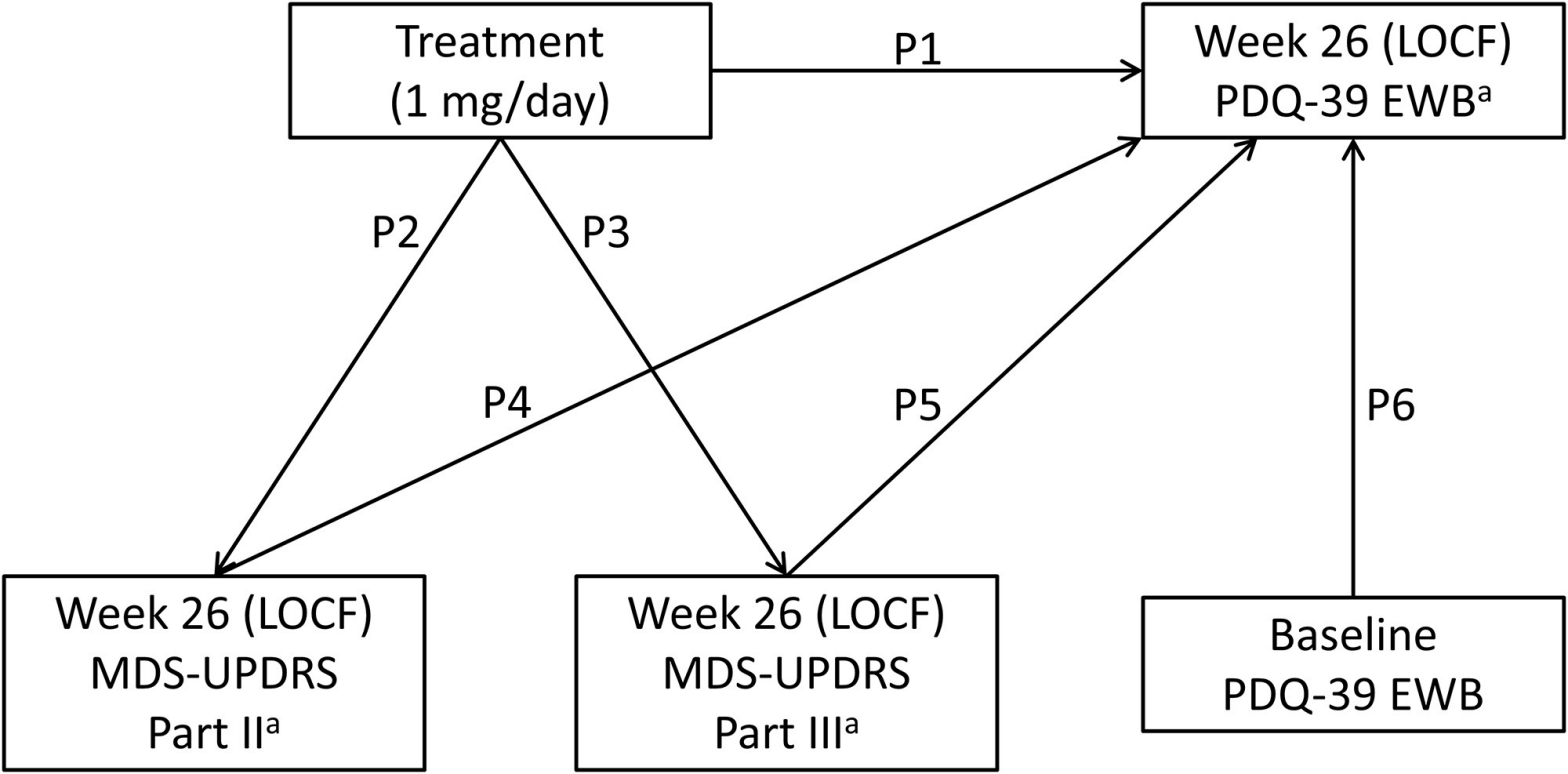
Hypothesized path diagrams in the monotherapy trial in early PD. P1 through P6 stand for the path coefficients (standardized regression coefficient) of each path. Covariances between exogeneous variables are not shown to aid in readability. ^a^Change from baseline. LOCF, last observation carried forward; MDS-UPDRS, Movement Disorder Society-Unified Parkinson’s Disease Rating Scale; PDQ-39 EWB, Emotional well-being domain of the 39-item Parkinson’s disease questionnaire.

**Fig 2:**
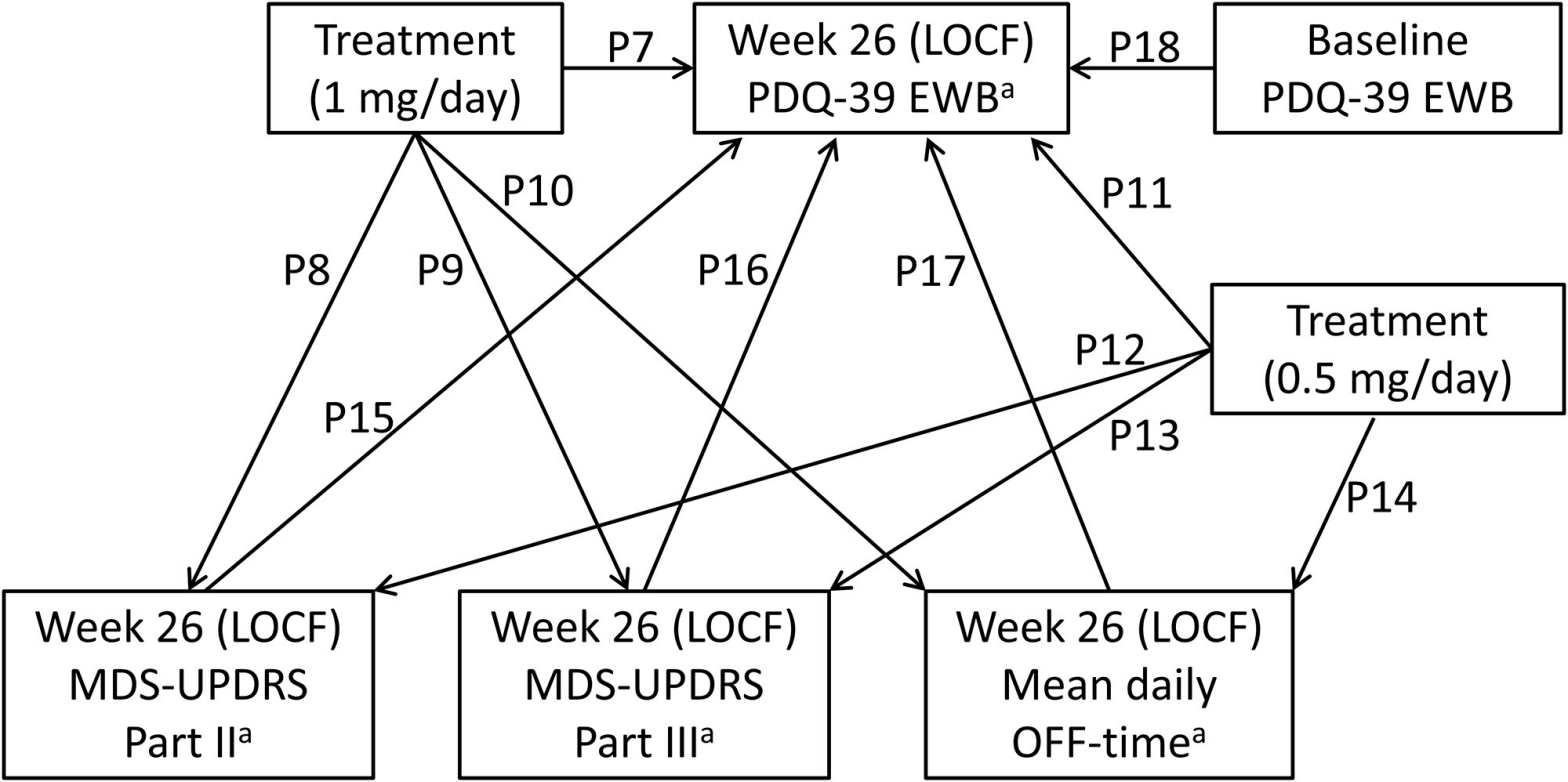
Hypothesized path diagrams in the adjunctive therapy trial in PD patients with wearing-off phenomena. P7 through P18 stand for the path coefficients (standardized regression coefficient) of each path. Covariances between exogeneous variables are not shown to aid in readability. ^a^Change from baseline. LOCF, last observation carried forward; MDS-UPDRS, Movement Disorder Society-Unified Parkinson’s Disease Rating Scale; PDQ-39 EWB, Emotional well-being domain of the 39-item Parkinson’s disease questionnaire.

A path analysis within a structural equation modeling framework using maximum likelihood estimation was performed to assess the direct treatment effects of rasagiline on the emotional well-being domain of PDQ-39 and the effects mediated indirectly through the influence on items related to motor symptoms. This methodology is used to determine the relative contribution of direct and indirect treatment effects of an intervention, and how they are mediated, in line with hypothesized underlying mechanisms [34]. Path diagrams adopted in this study are presented in Figs 1 and 2, depicting the hypothesized relationship between variables in a rasagiline monotherapy trial in early PD and an adjunctive therapy trial in patients with PD and wearing-off phenomena in Japan. The regression models used in each path diagram are shown in the (S1 Appendix). Single headed arrows represent the hypothesized causal order between two variables (the head pointing to the effect and the tail extending from the cause). Outcome (dependent) variables are referred to as endogenous variables, with every endogenous variable having at least one cause [35]. Some of these causes are independent variables, which are referred to as exogenous variables [35].

#### Selection of the variables

Three endogenous variables were selected for the monotherapy trial in early PD: change from baseline in MDS-UPDRS Part II score at week 26 (last observation carried forward [LOCF]); change from baseline in MDS-UPDRS Part III score at week 26 (LOCF); and change from baseline in PDQ-39 emotional well-being sub-score at week 26 (LOCF). Baseline PDQ-39 emotional well-being sub-score and randomized treatment (dummy variable: 1 = rasagiline 1 mg/day, 0 = placebo) were selected as exogenous variables.

Four endogenous variables were selected for the adjunctive therapy trial in PD patients with wearing-off phenomena: change from baseline in MDS-UPDRS Part II score at week 26 (LOCF); change from baseline in MDS-UPDRS Part III score at week 26 (LOCF); change from baseline in mean daily OFF-time at week 26 (LOCF); and change from baseline in PDQ-39 emotional well-being sub-score at week 26 (LOCF). Baseline PDQ-39 emotional well-being sub-score, randomized treatment 1 mg/day (dummy variable: 1 = rasagiline 1 mg/day, 0 = placebo or rasagiline 0.5 mg/day) and randomized treatment 0.5 mg/day (dummy variable: 1 = rasagiline 0.5 mg/day, 0 = placebo or rasagiline 1 mg/day) were selected as exogenous variables.

There were five major measures in the original trials—changes in MDS-UPDRS Part I, Part II, Part III, Part IV and mean daily OFF-time (adjunctive therapy trial only)—which could be potential mediators for the change in PDQ-39 emotional well-being sub-score in the path analysis from the perspective of clinical relevance. To estimate collinearity, an exploratory correlation analysis was conducted between potential mediators (The correlation matrices are in the [S1 Table]). Pearson correlation coefficients between the change in mean daily OFF-time and the change in MDS-UPDRS Part IV (r = 0.44; p<0.0001 in the adjunctive therapy trial) and between the change in MDS-UPDRS Part I and the change in MDS-UPDRS Part II (r = 0.43; p<0.0001 in monotherapy trial and r = 0.53; p<0.0001 in adjunctive therapy trial) were considered to be moderately strong, judging from both r >0.40 and statistical significance. Therefore, a decision was made to include only one of the measures in each pair in the path model in order to avoid multicollinearity. Between mean daily OFF-time and MDS-UPDRS Part IV, given the overlap between the two measures from a clinical perspective, the secondary endpoint of mean daily OFF-time was selected as a representative potential mediator of motor complications over the additional MDS-UPDRS Part IV endpoint. Between MDS-UPDRS Part I and Part II, MDS-UPDRS Part II was included because this study aimed to elucidate how the effect of rasagiline on PDQ-39 emotional well-being domain is mediated by the treatment effect of rasagiline on motor-related symptoms of PD, with the assumption that rasagiline’s direct contribution to PDQ-39 emotional well-being (through non-motor symptoms without being mediated by the effect on motor-related symptoms) should be estimated as direct effect in the path analysis where all the major motor-related variables—MDS-UPDRS Part II, Part III and mean daily-OFF time (only adjunctive therapy trial)—were included.

In this way, changes in MDS-UPDRS Parts II, MDS-UPDRS Part III and mean daily OFF-time (adjunctive therapy trial only)–items representing motor experiences of daily living, motor examination and motor complications, respectively–were chosen as potential mediators in the path analysis. Finally, the baseline PDQ-39 emotional well-being sub-score was included in order to control for effects of the baseline level on the change from baseline in PDQ-39 emotional well-being sub-score in both studies. These steps produced the final path model depicted in Figs 1 and 2.

#### Assumptions

Path analysis requires the assumptions of normal distributions and linear relationships among variables. The endogenous variables in the monotherapy and adjunctive therapy trials were considered to be normally distributed with univariate skewness values ranging from 0.04 to 0.81, and from -0.20 to 0.47, respectively and univariate kurtosis values ranging from 0.50 to 2.38, and from 1.68 to 2.61, respectively [35]. In addition, inspection of scatter diagrams between endogenous variables in each trial showed reasonably linear relationships. Therefore, the effects observed on PDQ-39 emotional well-being domain were assumed to be additive. Assumption of low multicollinearity was finally examined through tolerance and variance inflation factor (VIF). The tolerance ranging from 0.85 to 0.98, and from 0.74 to 0.98 (in the monotherapy and adjunctive therapy trial, respectively) and VIF index ranging from 1.02 to 1.18, and from 1.02 to 1.35 (in the monotherapy and adjunctive therapy trial, respectively) of all variables included in the models indicated this assumption was acceptable [35]. The covariances among all exogenous variables were set as free parameters following the customarily acceptable assumption [34]. The sample size of this study was safely assumed to be adequate according to the recommendation of Kline (2015) [35] considering there were at least 10 times as many observations as parameters in both trials (12 and 22 parameters, in the monotherapy trial and the adjunctive therapy trial, respectively). Error terms were assumed to be uncorrelated with any variable from the clinical perspective.

#### Model fit statistics

The Chi-square (χ^2^) statistic was used to assess the magnitude of discrepancy between sample and fitted covariance matrices with a non-significant test result indicating an adequate fit between the model and data [36]. Four other fit indices were also evaluated because the χ^2^ test is highly dependent on sample size [36, 37]. Both the Bentler’s Comparative Fit Index (CFI) and the Adjusted Goodness-of-Fit Index (AGFI) have ranges between 0 and 1, with a score of >0.95 indicating a good fit and scores >0.90 indicating an acceptable fit [38]. The root mean square error of approximation (RMSEA) estimates the lack of fit in a model compared with a perfect model; a value >0.1 indicates a poor fit, between 0.08 to 0.10 provides a mediocre fit, <0.08 indicates a reasonable fit, and <0.05 indicates a good fit [37, 38]. The standardized root mean square residual (SRMR) indicates a good model fit if the value is less than 0.08 [38].

Among all randomized patients who received at least one dose of study drug (the full analysis set), only those patients who had non-missing data for all variables depicted in Figs 1 and 2 were included in the path analysis.

All data analyses were performed using SAS version 9.4 (SAS Institute, Cary, NC, USA) and path analysis was conducted through the CALIS procedure.

## Results

### Path diagram evaluation

A total of 236 patients in the monotherapy trial in early PD and a total of 376 patients in the adjunctive therapy trial in patients with PD and wearing-off phenomena were evaluable for the present analysis (Table 2). There were 12 and 22 parameters in the monotherapy trial and the adjunctive therapy trial, respectively, thus the sample size for this study was deemed adequate according to the recommendation of Kline (2015) [35], given that the sample size was greater than a minimum of 10 times the number of parameters included in the path analysis models (19.7 and 17.1 times, in the monotherapy trial and the adjunctive therapy trial, respectively).

**Table 2:** Summary of model fit statistics.

	Monotherapy trial	Adjunctive therapy trial
	(n = 236)	(n = 376)
**χ**^**2**^ **(df)**	5.639 (3); p = 0.131	26.871 (6); p = 0.0002
**CFI**	0.975	0.902
**AGFI**	0.954	0.903
**SRMR**	0.040	0.054
**RMSEA (90% CI)**	0.061 (0.000–0.139)	0.096 (0.061–0.135)

AGFI, Adjusted Goodness-of-Fit Index; CI, confidence interval; CFI, Bentler’s Comparative Fit Index; df, degrees of freedom; PD, Parkinson’s disease; RMSEA, root mean square error of approximation; SRMR, standardized root mean square residual.

The path diagram of the monotherapy trial in early PD was considered to be appropriate after the conditions of the goodness-of-fit assessment were met (Table 2). The χ^2^ test of absolute fit was not significant (p = 0.13), indicating that this model was a good fit to the data. This conclusion was further supported by the RMSEA (<0.1), SRMR (<0.08), AGFI (>0.9) and CFI (>0.9) values.

The χ^2^ test of absolute fit for the path diagram of the adjunctive therapy study in patients with PD and wearing-off phenomena was statistically significant (p = 0.0002), suggesting that the model and data were not consistent. However, this model should not be rejected because the χ^2^ test was likely sensitive to the relatively large sample size in this study (Table 2) [36, 37]. This conclusion was supported by the RMSEA (<0.1), SRMR (<0.08), AGFI (>0.9), and CFI (>0.9) values indicating a reasonable fit for the path diagram, although the values of AGFI, RMSEA and CFI were borderline. Accordingly, the hypothesized path diagram for the adjunctive therapy trial in patients with wearing-off phenomena was considered to be reasonable overall.

### Path analysis in monotherapy trial in early PD

In the monotherapy trial in early PD, the PDQ-39 emotional well-being domain was predominantly affected by the indirect influence of items related to motor symptoms (MDS-UPDRS Part II and MDS-UPDRS Part III; Tables 3 and 4). The indirect effect of items related to motor symptoms (MDS-UPDRS Part II and MDS-UPDRS Part III) accounted for 80.7% of the total effect. Two-thirds (67.2%) of the total effect was mediated indirectly via the effect on MDS-UPDRS Part II, while 13.5% of the total effect was mediated indirectly via the effect on MDS-UPDRS Part III. The remainder (19.3%) of the total effect was attributed to a direct treatment effect of rasagiline.

**Table 3:** Estimated path coefficients for the hypothetical model in the monotherapy trial in early PD.

Path			Path coefficient^a^ (SE)		p-value
Treatment	→	Week 26 (LOCF)	P1	-0.0324 (0.0624)	0.6040
		PDQ-39 EWB			
Treatment	→	Week 26 (LOCF)	P2	-0.3113 (0.0589)	<0.0001
		MDS-UPDRS Part II			
Treatment	→	Week 26 (LOCF)	P3	-0.2657 (0.0606)	<0.0001
		MDS-UPDRS Part III			
Week 26 (LOCF) MDS-UPDRS Part II	→	Week 26 (LOCF)	P4	0.3628 (0.0567)	<0.0001
		PDQ-39 EWB			
Week 26 (LOCF) MDS-UPDRS Part III	→	Week 26 (LOCF)	P5	0.0853 (0.0589)	0.1479
		PDQ-39 EWB			
Baseline PDQ-39 EWB	→	Week 26 (LOCF)	P6	-0.3123 (0.0552)	<0.0001
		PDQ-39 EWB			

P1 through P6 are path coefficients depicted in Fig 1.

^a^Standardized regression coefficient representing the strength of the relationship between two variables. A path coefficient can range from -1.0 to 1.0, with higher numbers (positive or negative) indicating a stronger association.

LOCF, Last Observation Carried Forward; MDS-UPDRS, Movement Disorder Society-Unified Parkinson’s Disease Rating Scale; PD, Parkinson’s disease; PDQ-39 EWB, Emotional well-being domain of the 39-item Parkinson’s disease questionnaire; SE, standard error.

**Table 4:** Direct, indirect and total effect of rasagiline treatment on PDQ-39 emotional well-being domain in monotherapy trial in early PD.

	Standardized effect
	(Proportion of total effect)
**Direct effect**	-0.0324^a^ (19.3%)
**Indirect effect**	-0.1356 (80.7%)
Through MDS-UPDRS Part II	-0.1129^b^ (67.2%)
Through MDS-UPDRS Part III	-0.0227^b^ (13.5%)
**Total effect**	-0.1679^c^ (100.0%)

^a^Equal to P1 in Table 3.

^b^Calculated by the multiplying each path coefficient from treatment to the week 26 (LOCF) emotional well-being domain of PDQ-39. For example, indirect effect through MDS-UPDRS Part II was calculated by multiplying P2 by P4 in Table 3.

^c^Sum of the direct and indirect effects. Due to rounding, the total effect value does not exactly correspond with the sum of the values of the direct and indirect effects.

LOCF, Last Observation Carried Forward; MDS-UPDRS, Movement Disorder Society-Unified Parkinson’s Disease Rating Scale; PD, Parkinson’s disease; PDQ-39, 39-item Parkinson’s disease questionnaire.

### Path analysis in adjunctive therapy trial in patients with PD and wearing-off phenomena

In the adjunctive therapy trial in patients with PD and wearing-off phenomena, PDQ-39 emotional well-being domain was also mainly affected indirectly via the influence on items related to motor symptoms (MDS-UPDRS Part II, MDS-UPDRS Part III and mean daily OFF-time) accounting for 54.7% and 57.6% of the total effect for rasagiline 1 and 0.5 mg/day, respectively (Tables 5 and 6). MDS-UPDRS Part II mediated 35.6% and 40.9% of the total effect of rasagiline 1 and 0.5 mg/day, and MDS-UPDRS Part III mediated 8.0% and 8.3% of the total effect of rasagiline 1 and 0.5 mg/day, respectively. 11.1% and 8.4% of the total effect of rasagiline 1 and 0.5 mg/day was mediated indirectly via the reduction in mean daily OFF-time, respectively. The remainder (45.3% and 42.4%) of the total effect was attributed to the direct effect of rasagiline 1 and 0.5 mg/day, respectively, on the emotional well-being domain of PDQ-39.

**Table 5:** Estimated path coefficients for the hypothetical model in the adjunctive therapy trial in PD patients with wearing-off phenomena.

Path			Path coefficient^a^ (SE)		p-value
Treatment (1 mg/day)	→	Week 26 (LOCF)	P7	-0.0606 (0.0542)	0.2638
		PDQ-39 EWB			
Treatment (1 mg/day)	→	Week 26 (LOCF)	P8	-0.1490 (0.0575)	0.0096
		MDS-UPDRS Part II			
Treatment (1 mg/day)	→	Week 26 (LOCF)	P9	-0.1449 (0.0576)	0.0120
		MDS-UPDRS Part III			
Treatment (1 mg/day)	→	Week 26 (LOCF)	P10	-0.1719 (0.0573)	0.0027
		mean daily OFF-time			
Treatment (0.5 mg/day)	→	Week 26 (LOCF)	P11	-0.0443 (0.0536)	0.4084
		PDQ-39 EWB			
Treatment (0.5 mg/day)	→	Week 26 (LOCF)	P12	-0.1336 (0.0576)	0.0204
		MDS-UPDRS Part II			
Treatment (0.5 mg/day)	→	Week 26 (LOCF)	P13	-0.1172 (0.0578)	0.0426
		MDS-UPDRS Part III			
Treatment (0.5 mg/day)	→	Week 26 (LOCF)	P14	-0.1022 (0.0577)	0.0766
		mean daily OFF-time			
Week 26 (LOCF) MDS-UPDRS Part II	→	Week 26 (LOCF)	P15	0.3196 (0.0448)	<0.0001
		PDQ-39 EWB			
Week 26 (LOCF) MDS-UPDRS Part III	→	Week 26 (LOCF)	P16	0.0739 (0.0468)	0.1142
		PDQ-39 EWB			
Week 26 (LOCF) mean daily OFF-time	→	Week 26 (LOCF)	P17	0.0862 (0.0469)	0.0658
		PDQ-39 EWB			
Baseline PDQ-39 EWB	→	Week 26 (LOCF)	P18	-0.2478 (0.0453)	<0.0001
		PDQ-39 EWB			

^a^Standardized regression coefficient representing the strength of the relationship between two variables. A path coefficient can range from -1.0 to 1.0, with higher numbers (positive or negative) indicating a stronger association.

P7 through P18 are path coefficients depicted in Fig 2.

LOCF, Last Observation Carried Forward; MDS-UPDRS, Movement Disorder Society-Unified Parkinson’s Disease Rating Scale; PD, Parkinson’s disease; PDQ-39 EWB, Emotional well-being domain of the 39-item Parkinson’s disease questionnaire; SE, standard error.

**Table 6:** Direct, indirect and total effect of rasagiline treatment on PDQ-39 emotional well-being domain in adjunctive therapy trial in PD patients with wearing-off phenomena.

	Standardized effect (Proportion of total effect)	
	Rasagiline (1 mg/day)	Rasagiline (0.5 mg/day)
**Direct effect**	-0.0606^a^ (45.3%)	-0.0443^b^ (42.4%)
**Indirect effect**	-0.0731 (54.7%)	-0.0602 (57.6%)
Through MDS-UPDRS Part II	-0.0476^c^ (35.6%)	-0.0427 ^c^ (40.9%)
Through MDS-UPDRS Part III	-0.0107 ^c^ (8.0%)	-0.0087 ^c^ (8.3%)
Through mean daily OFF-time	-0.0148 ^c^ (11.1%)	-0.0088 ^c^ (8.4%)
**Total effect**	-0.1338 ^d^^,^ ^e^ (100.0%)	-0.1045 ^d^ (100.0%)

^a^Equal to P7 in Table 5.

^b^Equal to P11 in Table 5.

^c^Calculated by multiplying each path coefficient from treatment to the week 26 (LOCF) emotional well-being domain of PDQ-39. For example, indirect effect of rasagiline (1mg/day) through MDS-UPDRS Part II was calculated by multiplying P8 by P15 in Table 5.

^d^Sum of the direct and indirect effects.

^e^Due to rounding, the total effect value does not exactly correspond with the sum of the values of the direct and indirect effects.

LOCF, Last Observation Carried Forward; MDS-UPDRS, Movement Disorder Society-Unified Parkinson’s Disease Rating Scale; PD, Parkinson’s disease; PDQ-39, 39-item Parkinson’s disease questionnaire.

## Discussion

The treatment effect of rasagiline on the emotional well-being domain of PDQ-39 in Japanese patients with PD was predominantly mediated by the effect of rasagiline on motor-related items, especially through the influence of the MDS-UPDRS Part II (motor experiences of daily living) in both the monotherapy study in early PD and adjunctive therapy study in patients with PD and wearing-off phenomena. To our knowledge, this was the first study to evaluate the potential causal relationship between an individual pharmacological treatment and its outcomes assessed by the MDS-UPDRS utilizing path analysis, providing greater insight into the clinical benefits of rasagiline therapy for patients with PD.

While studies focusing on the relationship between individual pharmacological therapies and their outcomes utilizing path analysis have previously been investigated, these earlier studies were performed using data collected according to the original UPDRS scale [32, 39]. Although the UPDRS is a commonly used and validated scale for assessment of treatment efficacy in alleviating PD symptoms, the MDS-UPDRS is an updated and improved scale [33], which has been validated in Japanese patients with PD [11]. One of the major changes in the revision of the UPDRS scale was the adaptation of the questions in MDS-UPDRS Part II to be more appropriate as a self-reported questionnaire that can be completed independently by a patient and/or their caregiver [33]. However, the objective assessment of parkinsonism has been retained in MDS-UPDRS Part III [33].

### Motor-related items as mediators of emotional aspects of HRQoL

Given that HRQoL is a patient-reported outcome [12], it is logical that this study identified MDS-UPDRS Part II, which assesses motor experiences of daily living according to the patient, as the predominant contributor to the rasagiline-related effect on the emotional well-being domain of PDQ-39, rather than the objectively assessed MDS-UPDRS Part III.

The result reported here is also consistent with other studies that have suggested a lesser contribution of MDS-UPDRS Part III to HRQoL outcomes compared with MDS-UPDRS Part II [21, 22, 25, 26, 29, 30]. Likewise, several other studies using activities of daily living (ADL) scales other than MDS-UPDRS Part II, including the Schwab and England disability scale [27], the Short Parkinson’s Evaluation Scale/Scales for Outcomes in Parkinson’s disease (SPES/SCOPA) ADL section [28] and UPDRS Part II [23, 24], have also suggested that ADL has a greater impact on HRQoL than objectively assessed motor impairment, regardless of whether or not an ADL questionnaire is suitable for patient self-assessment. Therefore, alleviating symptoms related to activities and/or motor experiences associated with daily living may be expected to be translated to benefits in HRQoL, as reported in this study.

The comparatively lower contribution of mean daily OFF-time to HRQoL outcomes compared with motor experiences in daily living is also consistent with an earlier study that indicated motor complication items assessed by UPDRS Part IV [23, 24] or MDS-UPDRS Part IV [21, 22, 25, 26] had a relatively lower contribution to HRQoL than activities and/or motor experiences of daily living items, and that there was no statistically significant relationship between items related to motor fluctuations and the emotional well-being domain of the PDQ-39 [40, 41]. However, it must be noted that some studies have found a negative relationship between motor complications (dyskinesias and/or motor fluctuations) and HRQoL [40–45], but this may be a result of differences in inclusion/exclusion criteria, disease duration, treatment background, and methods of measuring HRQoL and motor complications between studies. Patients with severe dyskinesia were not included in our study due to the exclusion criteria of the rasagiline clinical trials in Japan [10], and the effect of dyskinesia was not taken into account in the path analysis because we adopted only mean daily OFF-time as the representative variable of motor complications; this represents a limitation of our study.

### Other possibilities and limitations

Although the PDQ-39 emotional well-being domain was mainly affected indirectly via the influence of rasagiline on items related to motor symptoms in both studies, the direct effect of rasagiline accounted for a greater proportion of the total effect in the adjunctive therapy study in patients with PD and wearing-off phenomena (1 mg/day: 45.3%, 0.5 mg/day: 42.4%) than in the monotherapy study in early PD (19.3%). However, contributions from other unidentified factors cannot be ruled out, especially in the adjunctive therapy study in patients with wearing-off phenomena because the pathophysiology and patient demographics in this study were considered to be more complex due to the longer duration of disease and concomitant treatments, which could have augmented the direct effect of rasagiline. Alternatively, or possibly in combination, rasagiline may have a greater direct effect on the emotional well-being domain of PDQ-39 in patients with advanced versus early PD, but a conclusive explanation cannot be drawn due to the post-hoc nature of this analysis, and the original trials not being designed to assess this question [9, 10].

Although the MDS-UPDRS Part II is used to evaluate motor-related symptoms, considering that this measure is a patient-reported outcome, which could be affected by the patient’s mood status, some part of the effect of rasagiline on this measure may be mediated by rasagiline’s direct contribution to the mood of the patients. In fact, the Pearson correlation coefficients between the change in MDS-UPDRS Part I and the change in MDS-UPDRS Part II in this study were moderately strong (r = 0.43 in monotherapy trial, r = 0.53 in adjunctive therapy trial) (S1 Table). In addition to that, some previous studies have suggested a direct role for rasagiline in depressive symptoms in PD patients [18] and some dopaminergic therapies were effective in treating depressive symptoms in the non-PD patients [46, 47]. Considering these, the possibility of rasagiline’s direct contribution to the effect on non-motor symptoms, especially for mood related symptoms, should not be excluded by the results obtained in this study.

While it was not investigated in this study, an interrelationship between MDS-UPDRS Part II, MDS-UPDRS Part III and mean daily OFF-time may exist. For example, previous path analysis studies demonstrated that objectively assessed motor impairment and motor complications influenced HRQoL indirectly through ADL in patients with PD [23, 28]. Accordingly, this study was limited by the interaction between MDS-UPDRS Part II, MDS-UPDRS Part III and mean daily OFF-time not being considered in this path analysis.

Despite this assessment being made in the context of prospective, randomized controlled trials, as opposed to earlier studies using observational, cross-sectional data, caution is required when attributing causality because all mediators and outcome variables were measured at week 26 (LOCF). Ideally, mediators would be assessed before outcomes.

In addition to the post-hoc nature of this analysis, as mentioned above, a generalization of these results is limited because the data included in this study were only from Japanese participants who met the inclusion criteria for the clinical trials of rasagiline in Japan [9, 10]. Applying these findings to PD patients with cognitive impairment and/or severe depression is also challenging, because patients with a Mini-Mental State Examination score ≤24, major or severe depression, and concomitant use of antidepressants were ineligible to participate in these studies [9, 10]. Accordingly, additional research is required to further understand the relevance of these limitations.

## Conclusions

Alleviating symptoms associated with motor experiences that affect daily living, which is subjectively assessed by the MDS-UPDRS Part II, was the predominant factor in rasagiline’s effect on stabilizing the emotional aspects of HRQoL in Japanese patients with PD, as assessed by PDQ-39 emotional well-being domain. Therefore, alleviating subjective motor-related symptoms in patients with PD should be considered as part of a pharmacological treatment plan for patients with PD to assist in managing the subjective emotional aspects of HRQoL.

## Supporting information

S1 Appendix Regression models. (DOCX)

S1 Table Correlation matrices. (DOCX)
