# Supplementary material for: Effects of rasagiline on Parkinson’s Disease Questionnaire (PDQ-39) emotional well-being domain in patients with Parkinson’s disease: A post-hoc analysis of clinical trials in Japan
Source: PLoS One. 2022 Jan 25;17(1):e0262796. doi: 10.1371/journal.pone.0262796 (PMC8789184; doi:10.1371/journal.pone.0262796)
Supplement: S1 Appendix — (DOCX) [file pone.0262796.s001.docx]

**S1 Appendix – Regression Models**

The regression models for the monotherapy trial in early PD (Fig. 1) were as follows:

ΔPart II = P2 * Treatment

ΔPart III = P3 * Treatment

ΔEWB = P1 * Treatment + P4 * ΔPart II + P5 * ΔPart III + P6 * EWB Baseline

In these equations, ΔPart II is the change from baseline in MDS-UPDRS Part II score at week 26 (LOCF); ΔPart III is the change from baseline in MDS-UPDRS Part III score at week 26 (LOCF); ΔEWB is the change from baseline in PDQ-39 emotional well-being sub-score at week 26 (LOCF); Treatment represents a dummy variable (1 = rasagiline 1 mg/day; 0 = placebo) and EWB Baseline indicates the baseline score of the PDQ-39 emotional well-being sub-score.

The total effect of treatment was determined by:

Total effect of treatment = P1 + P2 * P4 + P3 * P5

P1 through P6 in the above equation are the path coefficients (standardized regression coefficient) and P1 is the direct effect of treatment. The direct effect of treatment on PDQ-39 emotional well-being sub-score at week 26 (LOCF) was defined as the treatment effect remaining after controlling for changes in MDS-UPDRS Part II and MDS-UPDRS Part III.

The indirect effects were calculated by multiplying each path coefficient from treatment to the week 26 (LOCF) emotional well-being domain of PDQ-39 as shown below:

Indirect effect of treatment through MDS-UPDRS Part II = P2 * P4

Indirect effect of treatment through MDS-UPDRS Part III = P3 * P5

The regression models for the adjunctive therapy trial with wearing-off phenomena (Fig. 2) were as follows:

ΔPart II = P8 * Treatment (1 mg/day) + P12 * Treatment (0.5 mg/day)

ΔPart III = P9 * Treatment (1 mg/day) + P13 * Treatment (0.5 mg/day)

ΔOFF = P10 * Treatment (1 mg/day) + P14 * Treatment (0.5 mg/day)

ΔEWB = P7 * Treatment (1 mg/day) + P11 * Treatment (0.5 mg/day) + P15 * ΔPart II + P16 * ΔPart III + P17 * ΔOFF + P18 * EWB Baseline

In these equations, ΔPart II is the change from baseline in MDS-UPDRS Part II score at week 26 (LOCF); ΔPart III is the change from baseline in MDS-UPDRS Part III score at week 26 (LOCF); ΔOFF is the change from baseline in mean daily OFF-time at week 26 (LOCF); ΔEWB is the change from baseline in PDQ-39 emotional well-being sub-score at week 26 (LOCF); Treatment (1 mg/day) represents a dummy variable (1 = rasagiline 1 mg/day; 0 = placebo or rasagiline 0.5 mg/day); Treatment (0.5 mg/day) represents a dummy variable (1 = rasagiline 0.5 mg/day; 0 = placebo or rasagiline 1 mg/day) and EWB Baseline indicates the baseline score of the PDQ-39 emotional well-being sub-score.

The total effect of treatment was determined by:

Total effect of Treatment (1 mg/day) = P7 + P8 * P15 + P9 * P16 + P10 * P17

Total effect of Treatment (0.5 mg/day) = P11 + P12 * P15 + P13 * P16 + P14 * P17

P7 through P18 in the above equation are the path coefficients (standardized regression coefficient) and P7 and P11 are the direct effects of Treatment (1 mg/day) and Treatment (0.5 mg/day), respectively. The direct effect of treatment on PDQ-39 emotional well-being sub-score at week 26 (LOCF) was defined as the treatment effect remaining after controlling for changes in MDS-UPDRS Part II, MDS-UPDRS Part III and mean daily OFF-time.

The indirect effects were calculated by multiplying each path coefficient from treatment to the week 26 (LOCF) emotional well-being domain of PDQ-39 as shown below:

Indirect effect of Treatment (1 mg/day) through MDS-UPDRS Part II = P8 * P15

Indirect effect of Treatment (1 mg/day) through MDS-UPDRS Part III = P9 * P16

Indirect effect of Treatment (1 mg/day) through mean daily OFF-time = P10 * P17

Indirect effect of Treatment (0.5 mg/day) through MDS-UPDRS Part II = P12 * P15

Indirect effect of Treatment (0.5 mg/day) through MDS-UPDRS Part III = P13 * P16

Indirect effect of Treatment (0.5 mg/day) through mean daily OFF-time = P14 * P17
