# Supplementary material for: Effects of rasagiline on Parkinson’s Disease Questionnaire (PDQ-39) emotional well-being domain in patients with Parkinson’s disease: A post-hoc analysis of clinical trials in Japan
Source: PLoS One. 2022 Jan 25;17(1):e0262796. doi: 10.1371/journal.pone.0262796 (PMC8789184; doi:10.1371/journal.pone.0262796)
Supplement: S1 Table — (DOCX) [file pone.0262796.s002.docx]

**Supporting Information (S2 Correlation Matrices)**

1. **Pearson correlation matrix between potential mediators in the monotherapy trial**

|  | **1** | **2** | **3** |
| --- | --- | --- | --- |
| 1. **MDS-UPDRS Part I ^a^** | 1 |  |  |
| 1. **MDS-UPDRS Part II ^a^** | 0.43  (p<0.0001)^b^ | 1 |  |
| 1. **MDS-UPDRS Part III ^a^** | 0.24  (p=0.0002)^c^ | 0.21  (p=0.0014)^c^ | 1 |

^a^Change from Baseline at week 26 (LOCF). ^b^n=241. ^c^n=240.

MDS-UPDRS, Movement Disorder Society-Unified Parkinson’s Disease Rating Scale; LOCF, Last Observation Carried Forward.

1. **Pearson correlation matrix between potential mediators in the adjunctive therapy trial**

|  | **1** | **2** | **3** | **4** | **5** |
| --- | --- | --- | --- | --- | --- |
| 1. **MDS-UPDRS Part I ^a^** | 1 |  |  |  |  |
| 1. **MDS-UPDRS Part II ^a^** | 0.53  (p<0.0001)^b^ | 1 |  |  |  |
| 1. **MDS-UPDRS Part III ^a^** | 0.15  (p=0.0031)^c^ | 0.15  (p=0.0033)^c^ | 1 |  |  |
| 1. **MDS-UPDRS Part IV ^a^** | 0.16  (p=0.0019)^c^ | 0.20  (p<0.0001)^c^ | 0.18  (p=0.0003)^c^ | 1 |  |
| 1. **Mean daily OFF-time ^a^** | 0.15  (p=0.0024)^d^ | 0.12  (p=0.0178)^d^ | 0.18  (p=0.0006)^e^ | 0.44  (p<0.0001)^e^ | 1 |

^a^Change from Baseline at week 26 (LOCF). ^b^n=399. ^c^n=398. ^d^n=385. ^e^n=384.

MDS-UPDRS, Movement Disorder Society-Unified Parkinson’s Disease Rating Scale; LOCF, Last Observation Carried Forward.
